# Breaking the Mould: How the First Structure of a Deer Prion Suggests the Framework for Interspecies Strain Diversity and Transmission Barriers

**DOI:** 10.1111/jnc.70050

**Published:** 2025-03-25

**Authors:** Szymon W. Manka

**Affiliations:** ^1^ Department of Infectious Disease Imperial College London London UK

## Abstract

Our Insight into the Structural Diversity of Prions Has Been Limited by Studies Focused on Rodent‐Adapted Sheep (Scrapie) Prion Strains, Until Now. In a Recent Paper (Alam et al. 2024), the Caughey Research Group Presents the First Prion Structure from a Naturally Occurring Chronic Wasting Disease (CWD), Offering a Fresh Perspective.

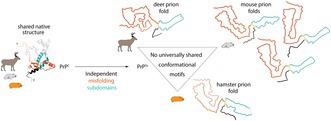

## Graphical abstract

The normal prion protein (PrP^C^) fold is shared among mammals; however, it can undergo misfolding in various ways, giving rise to distinct infectious prion (PrP^Sc^) strains. This review describes independent misfolding subdomains within PrP, recognised through new structural alignments and comparisons with the novel deer chronic wasting disease (CWD) prion structure. It shows that these conformationally independent segments support prion diversity, lacking any universally conserved conformational motifs across various host species. It further suggests that certain species‐specific conformations may be implicated in interspecies prion transmission barriers.

**Abbreviations**
ad
: Alzheimer's disease
Aβ: amyloid‐β
BSE: bovine spongiform encephalopathy
CJD: Creutzfeldt‐Jakob disease
C‐lobe: C‐terminal lobe of PrP^Sc^
Cryo‐EM: electron cryo‐microscopy
CWD: chronic wasting disease
DS: disulphide‐stapled
GPI: glycosylphosphatidylinositol
N‐lobe: N‐terminal lobe of PrP^Sc^
NMR: nuclear magnetic resonance
PD: Parkinson's disease
PIRIBS: parallel in‐register intermolecular β‐sheet
PrP: prion protein
PrP^C^
: cellular prion protein
PrP^Sc^
: scrapie prion protein
TSE: transmissible spongiform encephalopathy
vCJD: variant Creutzfeldt‐Jakob disease

## 1 Prion Diseases and Their Wider Relevance

Prions are protein‐only infectious pathogens causing untreatable and invariably fatal neurodegeneration in mammals (Prusiner 1982, 1998). Prion diseases, or transmissible spongiform encephalopathies (TSEs), include Creutzfeldt‐Jakob disease (CJD) in humans, scrapie in sheep and goats, bovine spongiform encephalopathy (BSE or ‘mad cow’ disease) in cattle and chronic wasting disease (CWD or ‘zombie deer’ disease) in cervids (Collinge 2016). During the UK outbreak in the 1990s, millions of cows were infected with BSE prions, which crossed over to humans through ingestion of contaminated bovine food products, causing variant CJD (vCJD) (Collinge et al. 1996). CWD, on the other hand, is a uniquely contagious TSE (Miller and Williams 2004). It can spread via body fluids and excreta, while decomposing ‘zombie deer’ carcasses contaminate soil and water. As a result, CWD has become endemic among captive and farmed cervids on several continents (Bartz et al. 2024; EFSA Panel on Biological Hazards (BIOHAZ) et al. 2017), raising concerns about its potential zoonotic transmission through consumption of hunted or farmed deer or elk meat or in a contagious manner. The disease is extremely difficult to eradicate, as prions are highly resistant to heat and conventional sterilisation methods. Thus, animal prions are a threat to humans, and understanding the structural basis of their infectivity and pathogenicity is important. Moreover, prion disease is increasingly recognised as a paradigm for the commoner dementias, such as Alzheimer's disease (ad) and Parkinson's disease (PD), which impose an enormous burden on society. Significantly, all these neuropathies are also proteinopathies, as they share a central feature involving the formation of multichain ribbon‐like (amyloid) fibrils in the brain (Goedert 2015). The mounting evidence of the transmissibility of these inclusions via prion‐like mechanisms (Banerjee et al. 2022, 2024) has lent credence to classifying these disorders as true prion diseases (Condello et al. 2024). Consequently, their respective proteopathic seeds are considered distinct types of prions.

## 2 Prion or Amyloid, Which Concept Is Expanding?

**FIGURE 1:**
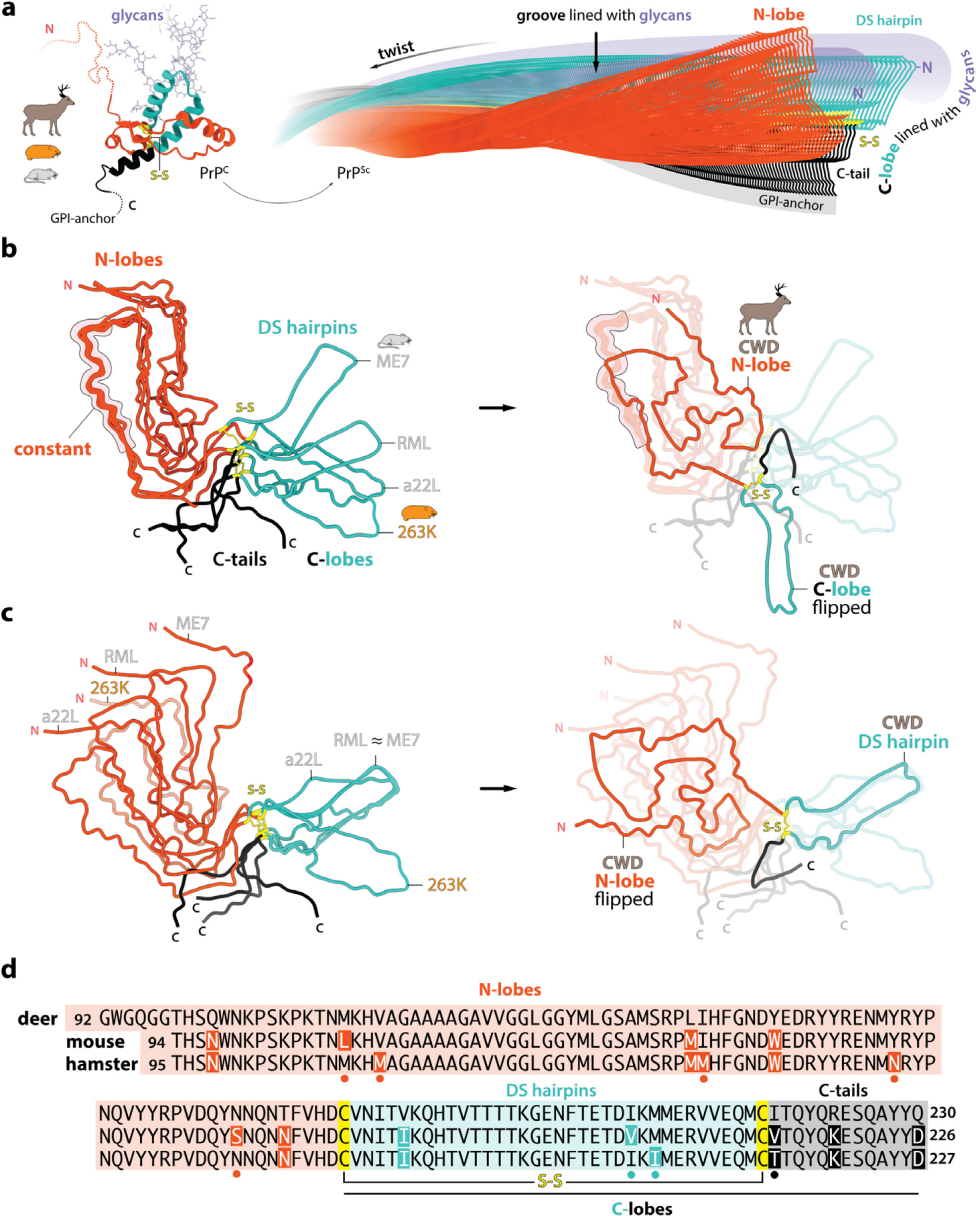
Prions lack universally conserved conformational motifs across different host species. (a) Left, superposed cellular prion protein (PrP^C^) tertiary structures from: Deer (pdb code: 4YXH) (Baral et al. 2015), hamster (pdb code: 4YXL) (Baral et al. 2015) and mouse (pdb code: 4H88) (Sonati et al. 2013) colour‐coded according to boundaries of misfolding subdomains separated by the disulphide (S‐S) bridge. Flexible N‐terminal (N) and C‐terminal (C) regions are indicated with dotted lines. C‐terminal glycosylphosphatidylinositol membrane anchor (GPI‐anchor), S‐S bridge and example asparagine (N)‐linked glycans are indicated. Right, an example 3D model of a wild‐type prion fibril with indicated common characteristics. The N‐lobe and disulphide‐stapled (DS) hairpin form independently on either side of the S‐S bridge. The DS hairpin and the C‐tail together form the C‐lobe. The positions of ‘fuzzy’ densities of flexible N‐linked glycans (‐N) in the inter‐lobe groove and at the tip of the DS hairpin and the position of the GPI‐anchor are indicated. β‐sheets or lateral intra‐ and inter‐chain (interlocking) interactions are not depicted. (b, c) Left, single‐chain comparisons of PrP folds from mouse prion strains: RML (pdb code: 7QIG) (Manka, Zhang, et al. 2022), ME7 (pdb code: 8A00) (Manka et al. 2023) and a22L (pdb code: 8EFU) (Hoyt, Alam, et al. 2022; Hoyt, Standke, et al. 2022), and hamster prion strain 263K (pdb code: 7LNA) (Kraus et al. 2021), aligned on the conformationally conserved (constant, silhouetted) region identified in rodent prions (b) or on the S‐S bridge region (c). Right, the same alignments (dimmed) with accordingly overlaid novel PrP fold from deer (CWD) prion strain (pdb code: 9DMY) (Alam et al. 2024). N, N‐terminus; C, C‐terminus; ≈, similar. (d) Alignment of deer, mouse and hamster PrP sequences mapping to protease‐resistant prion fibril cores. Deer and rodent sequence mismatches are highlighted directly in rodent sequences, and mouse and hamster mismatches are indicated with dots. Deer v. mouse: 11 mismatches, deer v. hamster: 12 mismatches, mouse v. hamster: 8 mismatches.

The prion principle is founded on the transmissible pathology of a 27–30 kDa cell surface glycoprotein named prion protein (PrP) (Prusiner et al. 1984). The N‐terminal roughly half of native cellular PrP (PrP^C^) is disordered, while the C‐terminal counterpart is an ⍺‐helix‐rich domain glycosylphosphatidylinositol (GPI)‐anchored to the cell membrane (Figure 1a, Left). That globular C‐terminal domain undergoes a dramatic conformational change to form a filamentous prion assembly, designated as PrP^Sc^ (Meyer et al. 1986; Prusiner 1998) (Figure 1a, Right). X‐ray or NMR structures of PrP^C^ from various mammalian host species have been known for decades. Remarkably, they all share a near‐identical PrP fold despite differences in the amino acid sequence (Baral et al. 2015; Lysek et al. 2005) (Figure 1a, Left). However, the atomic‐level PrP^Sc^ structure remained unknown until relatively recently. Many expected it to be fundamentally unique, in line with the unparalleled infectivity and lethality of classic prions, but electron cryo‐microscopy (cryo‐EM) structures of rodent‐adapted scrapie prion fibrils derived from terminally infected hamster or mouse brains showed that prion fibrils were amyloids (Kraus et al. 2021; Hoyt, Alam, et al. 2022; Hoyt, Standke, et al. 2022; Manka, Wenborn, et al. 2022; Manka, Zhang, et al. 2022; Manka et al. 2023), just like tau or amyloid‐β (Aβ) filaments in ad or ⍺‐synuclein fibrils in PD (Fitzpatrick et al. 2017; Yang, Arseni, et al. 2022; Yang, Shi, et al. 2022). The defining parallel in‐register intermolecular β‐sheet (PIRIBS) structural arrangement provides the ordered, protease‐resistant fibril core. This has now been confirmed with the first natural CWD prion fibril structure from deer (Alam et al. 2024). So, all prions appear to be amyloids, but should the reverse be said of all transmissible amyloids? Classic prions appear structurally distinct due to their sugar coat and GPI anchoring to the cell surface—features likely responsible for their notorious infectivity (invasiveness and resistance to clearance), toxicity, and the resulting rapid lethality after disease onset. Furthermore, TSEs, with their highly distinctive spongiform pathology, are uniquely present across mammals and can even be transmitted across species. This combination of exceptional properties may warrant restricting the term ‘prion’ to PrP pathogens and justify using ‘prion‐like’ for other proteopathic seeds associated with milder (slowly progressing) clinical conditions in humans. Yet, milder does not mean simpler to cure. Current research suggests that PrP is not essential for brain function, raising the possibility that blocking its expression could cure prion disease. Thus, when a safe method to lower PrP levels in the brain becomes available, prion disease might be eliminated. The pathophysiological profiles of ad or PD appear much more complex from the therapeutic perspective, highlighting the potential risks of oversimplifying the shared mechanisms of amyloidogenesis. Here, the classical meaning of prions will be maintained.

## 3 Prion Strains Are Encoded in Shape

Prion formation can be spontaneous (sporadic) or seeded by infection, with propagation driven by a cycle of templated aggregation and fission. The templating provides the mechanism for prion strain replication, while potential imperfections in this process would lead to strain mutation. Distinct prion strains—operationally defined by a heritable phenotype of disease under controlled experimental conditions (DeFranco et al. 2024; Gunnels et al. 2023)—are, therefore, encoded by distinct conformations of PrP^Sc^ (Hoyt, Alam, et al. 2022; Hoyt, Standke, et al. 2022; Manka et al. 2023; Manka, Wenborn, et al. 2022; Manka, Zhang, et al. 2022). Crucially, even PrP monomers of the same primary structure can produce different PrP^Sc^ folds (intra‐species strains) (Hoyt, Alam, et al. 2022; Hoyt, Standke, et al. 2022; Manka et al. 2023). The stacking of PrP chains can stabilise those alternative conformations or folds through longitudinal hydrogen bonding arrays (inter‐chain β‐sheets) and by lateral intra‐ and inter‐chain (interlocking) hydrophobic and ionic interactions (Manka, Wenborn, et al. 2022; Manka, Zhang, et al. 2022), resulting in fibrils with defined shape and helical twist (Figure 1a, Right). This aligns with findings that α‐synuclein filaments from PD‐related disorders feature a unique structure, termed the Lewy fold, which differs notably from those found in another synucleinopathy, Multiple System Atrophy (Yang, Shi, et al. 2022).

A variety of strains and strain transmission barriers emerge through variations in host PrP sequence (between‐ and within‐species with sequence polymorphisms) and in conformational plasticity of each sequence variant. This diversity may also be influenced by as yet unidentified protein folding co‐factors acting at various extra‐ or intra‐cellular niches, and by different PrP glycoforms found at defined ratios in PrP^Sc^ isolates from different prion strains (biochemical strain signatures) (Owen et al. 2007; Rudd et al. 1999). Whilst fuzzy densities representing flexible glycans and the GPI‐anchor are straightforward to locate in cryo‐EM maps of prion fibrils, other extra non‐protein densities, which may represent unique misfolding co‐factors, are difficult to assign. So far, orthogonal techniques including mass spectrometry have not provided any clues.

## 4 It Takes Two to Strike, but Each Makes Its Own Move: Independent Misfolding Subdomains in PrP Provide the Framework for Species‐Specific Strain Diversity

Structures of rodent‐adapted scrapie prions (hamster 263K strain (Kraus et al. 2021) and mouse RML (Manka, Wenborn, et al. 2022; Manka, Zhang, et al. 2022), ME7 (Manka et al. 2023) and a22L (Hoyt, Alam, et al. 2022; Hoyt, Standke, et al. 2022) strains) determined by cryo‐EM to date consistently identified two‐lobed protease‐resistant fibril cores, composed of the C‐terminal approximately two‐thirds of the PrP chain (Figure 1b–d). The first cryo‐EM structure of a natural CWD prion retains this property, but in the CWD fold the two lobes are flipped relative to rodent prion folds (Figure 1b,c). The swivel point coincides with the first cysteine of the disulphide bridge, which should thus be considered the lobe boundary residue. Accordingly, the N‐terminal lobe (N‐lobe) extends from the start of the ordered fibril core to the first cysteine of the disulphide bridge, and the C‐terminal lobe (C‐lobe) consists of the disulphide‐stapled (DS) hairpin and the C‐tail (Figure 1a–d).

Notably, when aligned on the N‐lobe's region recognised as conformationally conserved among rodent‐adapted strains (Manka et al. 2023), the novel CWD fold stands out for not conforming (Figure 1b). An alignment focused on the disulphide bridge, which joins together the three universally present segments (N‐lobes, DS hairpins and C‐tails), indicates how the disulphide bridge may act as a conformational anchor, letting the N‐lobe and DS hairpin to form independently of each other, while each can be influenced by interactions with the C‐tail (Figure 1c). The position of the C‐tail can thus affect the inter‐lobe angle (the groove size, Figure 1a, Right) and chain planarity (the extent of PrP monomer interlocking along the fibril axis). With this alignment, we also appreciate a close similarity between DS hairpins of the deer CWD and mouse RML and ME7 folds (Manka et al. 2023) (Figure 1c). This shows that certain conformational motifs can be shared by prions from different host species, even if not conserved within the same species (different in a22L (Hoyt, Alam, et al. 2022).

## 5 What Can the Novel Prion Structure Teach Us About Cross‐Species Barriers to Prion Spread?

More prion structures from various species are needed to fully grasp the diversity of prion folds. However, it can be expected that the basic two‐lobed architecture will be preserved across hosts, generating protease‐resistant prion fibril cores spanning the C‐terminal two‐thirds of the PrP sequence (Figure 1d). Moreover, multi‐layered and heavily interlocked N‐lobes will likely be more conformationally conserved than predominantly two‐layered and relatively planar C‐lobes. The overall shapes of N‐lobes may become signatures of the host species, while a range of C‐lobe shapes might distinguish strains. This plasticity of C‐lobes may also underlie the prion ensemble (quasispecies) phenomenon, where dominant *strains* (i.e., conformational variants) coexist with minor *strains* (Gunnels et al. 2023), which may overcome certain interspecies transmission barriers, expanding the potential host ranges of such polyclonal strains (Steadman et al. 2024). Alternatively, the unique lobe configuration of the only deer prion fold known so far (Alam et al. 2024) indicates how certain transmission barriers might be effectively insurmountable for certain conformers.

Mapping PrP sequence variants onto known prion structures (templates) could aid in predicting the strength of corresponding transmission barriers (including zoonotic potential). For instance, if a destructive (incompatible with the current template fold) amino acid mismatch occurs within a three‐layered, interlocked core of an N‐lobe, the transmission barrier will likely be robust, but if it occurs in an inherently wobbly C‐lobe, adaptation to a new host may be more feasible (adaptive plasticity). Nonetheless, minor co‐infecting variants could still drive transmission (Steadman et al. 2024), and these may be difficult to detect with cryo‐EM. Notably, the consensus 3D reconstruction of the CWD prion fibril (Alam et al. 2024) shows extra densities around the C‐lobe, which may indeed represent minor alternative conformations of the DS hairpin, mixed into the density of the dominant strain.

## 6 Conclusions and Outlook

Although we have only begun to uncover the repertoire of the thermodynamically permissible prion folds in different hosts (Collinge and Clarke 2007), we already know that there are no universally conserved conformational motifs in prions (Alam et al. 2024). We have yet to see if species‐specific (or genetic polymorph‐specific) substructures generally exist and if they play a role in transmission barriers. Given the evidently strong barrier for their transmission to humans, the existing sheep, rodent‐adapted or cervid (CWD) prions are unlikely to share N‐lobe conformations with CJD or BSE prions that template the conversion of human PrP. It is challenging to verify this experimentally (using cryo‐EM) due to safety concerns.

The key question is whether the CWD prions could become zoonotic. Presently, there is no evidence of transmission in humanised transgenic mice (Groveman et al. 2024; Wadsworth et al. 2022). However, the total number of prion strains comprising CWD is unknown. Cervid PrP polymorphisms and the diversity of the genetic background of host and potential vector animal species provide an ample basis for strain selection and evolution. Testing the transmission properties of a greater range of CWD isolates from multiple cervid species is needed, and cryo‐EM experiments should not be attempted prior to tests in humanised mice.

In conclusion, the risk of a contagious CWD‐like neurodegenerative disease in people appears low, but vigilance and continued research into prion diseases is important to ensure preparedness for a potential prion epidemic that may be of far greater magnitude than the ‘mad cow’ disease crisis in the UK during the 1990s.

## Author Contributions

**Szymon W. Manka:** writing – original draft, writing – review and editing, visualization, formal analysis, funding acquisition.

## Conflicts of Interest

The author declares no conflicts of interest.

### Peer Review

The peer review history for this article is available at https://www.webofscience.com/api/gateway/wos/peer‐review/10.1111/jnc.70050.

## Data Availability

Data sharing is not applicable since no new data were generated for this manuscript.
